# Mechanism and Characteristics of Humidity Sensing with Polyvinyl Alcohol-Coated Fiber Surface Plasmon Resonance Sensor

**DOI:** 10.3390/s18072029

**Published:** 2018-06-25

**Authors:** Yu Shao, Ying Wang, Shaoqing Cao, Yijian Huang, Longfei Zhang, Feng Zhang, Changrui Liao, Yiping Wang

**Affiliations:** Key Laboratory of Optoelectronic Devices and Systems of Ministry of Education and Guangdong Province, College of Optoelectronic Engineering, Shenzhen University, Shenzhen 518060, China; shaoyu2016@email.szu.edu.cn (Y.S.); caoshaoqing@email.szu.edu.cn (S.C.); huangyijian@email.szu.edu.cn (Y.H.); zhanglongfei2016@email.szu.edu.cn (L.Z.); zhangfeng@szu.edu.cn (F.Z.); cliao@szu.edu.cn (C.L.); ypwang@szu.edu.cn (Y.W.)

**Keywords:** fiber optics sensors, relative humidity, polyvinyl alcohol, breathing test

## Abstract

A surface plasmon resonance (SPR) sensor based on a side-polished single mode fiber coated with polyvinyl alcohol (PVA) is demonstrated for relative humidity (RH) sensing. The SPR sensor exhibits a resonant dip in the transmission spectrum in ambient air after PVA film coating, and the resonant wavelength shifts to longer wavelengths as the thickness of the PVA film increases. When RH changes, the resonant dip of the sensor with different film-thicknesses exhibits interesting characteristics for optical spectrum evolution. For sensors with initial wavelengths between 550 nm and 750 nm, the resonant dip shifts to longer wavelengths with increasing RH. The averaged sensitivity increases firstly and then drops, and shows a maximal sensitivity of 1.01 nm/RH%. Once the initial wavelength of the SPR sensor exceeds 850 nm, an inflection point of the resonant wavelength shift can be observed with RH increasing, and the resonant dip shifts to shorter wavelengths for RH values exceeding this point, and sensitivity as high as −4.97 nm/RH% can be obtained in the experiment. The sensor is expected to have potential applications in highly sensitive and cost effective humidity sensing.

## 1. Introduction

The monitoring of relative humidity (RH) of the environment around the analyte is critical in a variety of situations such as food fabrication, medical research, agricultural production, weather prediction, and air quality monitoring. Therefore, the design of a highly sensitive RH sensor is of great interest and has been developed continuously in the applications mentioned above. Compared to a traditional electric RH sensor, optical fiber-based sensors exhibit a great number of advantages, such as security to the human body, miniaturization for integration, and good resistance to electromagnetic interference. In recent years, a variety of fiber devices have been implemented in to the design of optical fiber RH sensors, configurations including interferometers [1,2,3,4,5,6,7], long period fiber gratings [8,9], tapered fibers [10,11,12,13], fiber Bragg gratings (FBGs) [14,15,16,17,18], mode couplers [19,20], ZnO nanostructures [21,22], evanescent wave absorption structures [23,24], and so on. Among these configurations, light intensity demodulation is often used since it is simple and cost effective. For example, Y. Miao et al. have demonstrated a RH sensor based on a polyvinyl alcohol (PVA) coated tilted FBG, of which the sensitivities are 2.52 and 14.95 dBm/RH% under RH of 20–74% and 74–98%, respectively [18]. However, the stability and accuracy of intensity demodulation techniques are generally disturbed by system noises and the fluctuation of light source. Thus, there are also choices that utilizing wavelength demodulation techniques to overcome the problems existed in intensity demodulations.

PVA is a frequently used hygroscopic material with excellent optical properties for the construction of wavelength demodulated optical fiber RH sensors. It can be easily dissolved in hot water and coated onto a fiber surface by a dip-coating treatment. When exposed in a humid environment, the refractive index (RI) of PVA can be changed dramatically by the surrounding RH, based on which many fiber RH sensors have been proposed and demonstrated. Wong et al. proposed a PVA coated PCF sensor to measure the environmental RH and the sensitivity reaches 0.60 nm/RH% in the RH range from 30% to 90% [3]. Zamarreno et al. reported a multimode fiber sensor for RH measurement with a much higher sensitivity of 1.2 RH% per nm [25]. The sensor was fabricated by coating 100 layers of ITO films and exhibits a very broad resonance peak. Therefore, there is still room to improve device performance, such as the simplification of device fabrication, reduction of the full-width at half-maximum resonance, and the enhancement of RH sensitivity. To overcome the abovementioned problems, surface plasmon resonance (SPR) can be considered in the design of novel humidity sensors. K. Shinbo et al. has designed an experiment where a PVA film coated SPR device was employed and the transient shift of the SPR wavelength under a humidity level of 95% was recorded, and a red-shift followed by a blue-shift of the SPR wavelength was observed with increasing time, which indicated the existence of a competition effect between the thickness expansion and the RI reduction of the PVA films [26]. However, competition results in a SPR configuration for PVA films under different steady states of humidity levels have not been studied, which are meaningful for the design of PVA-based humidity sensors.

In this paper, we present a simple realization of a PVA-coated fiber SPR sensor for RH measurement and demonstrate the competition effect between the thickness expansion and RI reduction of the PVA film under various steady state humidity levels. The sensor is fabricated by coating a very thin layer of PVA film onto the Au-surface of a side-polished single mode fiber (SMF) SPR device. The resonant wavelength of the sensor in ambient air is mainly determined by the thickness of the PVA film. When the initial wavelength increases from 550 nm to 750 nm, the resonant dip shifts to longer wavelengths and the averaged sensitivity increases from 0.37 nm/RH% to a maximum of 1.01 nm/RH% (with initial wavelength of 650 nm), then drops to 0.45 nm/RH%. For sensors with initial wavelengths beyond 850 nm, an inflection point of the resonant dip shift can be observed with increasing RH; i.e., the resonant dip shifts to shorter wavelengths for RH exceeding this point. And in this case, a sensitivity as high as −4.97 nm/RH% can be obtained for ambient humidity between 80% and 90%. The sensor is expected to be a good candidate for humidity sensing applications.

## 2. Materials and Methods

### 2.1. SPR Sensor Fabrication

A standard SMF with a core diameter of 8.2 μm and a cladding diameter of 125 μm was used to fabricate the fiber SPR sensor. The SMF was side-polished to a D-shaped geometry by a fiber polishing system that we reported previously [27]. The thickness and length of the side-polished area were 70 ± 0.8 μm and 5 mm, respectively. Then, a gold film with a thickness of 55 nm was deposited onto the flat surface of the D-shaped fiber by magnetron sputtering technique [28]. Here the obtained sensor exhibited no resonant dips in the transmission spectrum unless it was immersed into liquids with RI lower than that of the fiber core.

### 2.2. Polyvinyl Alcohol Coating

Aqueous PVA solutions with concentrations from 0.5% to 5% (wt/wt) were prepared by dissolving PVA particles with deionized water, using a magnetic stirrer at 90 °C for 1 h, and the fabricated SPR sensor was immersed into one of these solutions and coated with PVA by the dip-coating technique, with a constant withdrawal speed of 4 cm/s. Finally, a homogeneous PVA film with a thickness between 60 nm and 400 nm (measured by step profiler) was formed after drying for 2 h, and a resonant dip was found between 550 nm and 750 nm in the transmission spectrum of the sensor in ambient air. Moreover, by repeating the processes of dip-coating and drying, the thickness of the PVA film could be increased further and thus the initial wavelength of the SPR sensor could be tuned to longer wavelengths effectively.

### 2.3. Experimental Setup

**Figure 1:**
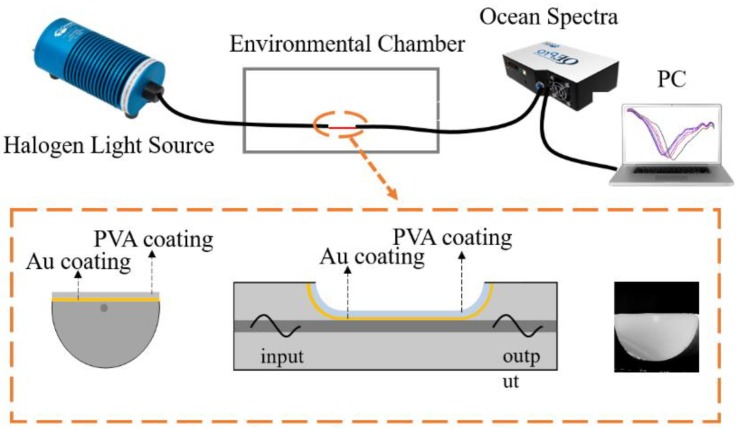
Schematic diagram of the relative humidity (RH) sensing system. Lower insets show the geometries in cross-section view, side view, and the microscopic image in cross-section view of the polyvinyl alcohol (PVA) coated surface plasmon resonance (SPR) sensor, respectively.

To investigate the response to RH, the fiber SPR sensor was placed in a homemade RH chamber, with one end connected with a halogen light source (400–2000 nm, Ocean Optics) and the other end connected with a fiber spectrometer (Ocean Optics), respectively, as shown in Figure 1. In the RH chamber, there was a humidifier and drier for increasing and decreasing RH, respectively, a fan for homogenization and stabilization, and an electric hygrometer for calibration. The RH could be controlled from 40% to 90% with an accuracy of 0.1%. The transmission spectra of the sensor were collected by the spectrometer and recorded and post-processed by a personal computer (PC) in real-time during the test. Meanwhile, the RH data collected by the electric hygrometer for calibration were also recorded by the PC simultaneously.

## 3. Results and Discussion

### 3.1. Relative Humidity Response

**Figure 2:**
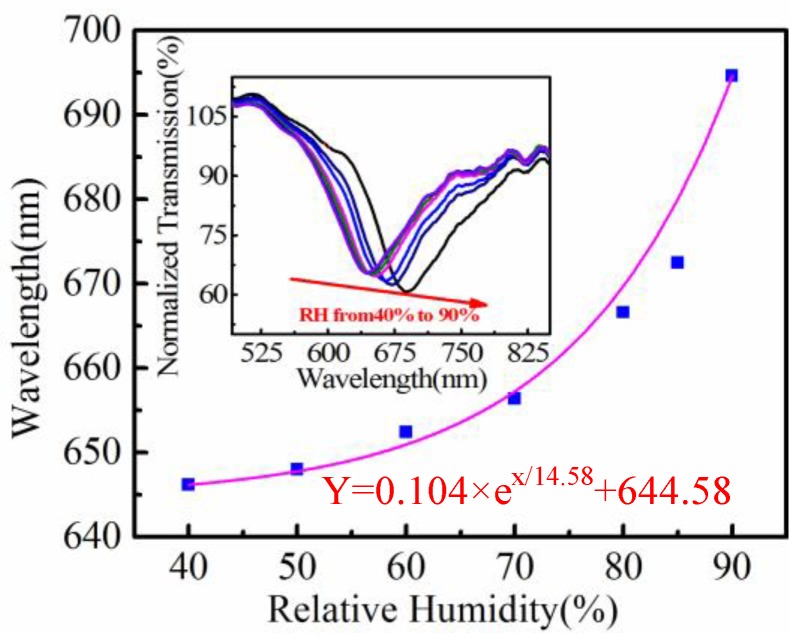
Resonant wavelength shift and spectrum evolution of a fiber SPR sensor with RH increasing. The initial wavelength is 645.52 nm, and the purple curve is an exponential fitting to the experimental data.

Figure 2 shows the resonant wavelength shift of a SPR sensor with an initial wavelength near 650 nm in ambient air (fabricated by dip-coating with a 3% (wt/wt) PVA solution). The resonant dip shifts from 645.52 nm to 695.94 nm with increased RH from 40% to 90%. Note that the temperature in the chamber is kept constant to avoid temperature cross-sensitivity. For RH between 40% and 70%, the resonant wavelength of the sensor shifts to longer wavelengths nearly linearly with a sensitivity of ~0.36 nm/RH%. However, the sensitivity increases rapidly for RH from 70% to 90%, and achieves 4.69 nm/RH% for that between 85% and 90%. The averaged sensitivity of the sensor can be roughly estimated to be 1.01 nm/RH% in the RH range between 40% and 90%. Actually, the dip wavelength shift can be more appropriately fitted with an exponential function as shown in Figure 2. The total wavelength shift of the sensor is 50.42 nm, which is comparable to that of the most sensitive fiber RH sensor reported so far [25].

**Figure 3:**
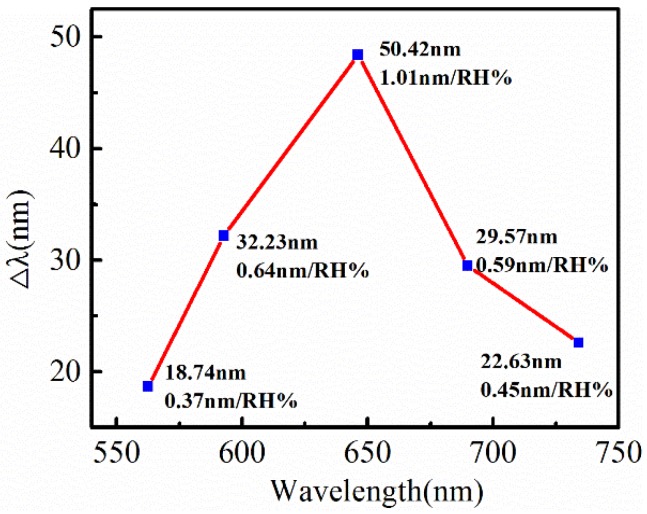
Averaged RH sensitivities of the fiber SPR sensor with different initial wavelengths (or thicknesses of the PVA film).

We found that RH sensitivities of the fiber SPR sensors were quite different with the initial changes in wavelength (or the thickness of the PVA film). Five sensors with initial wavelengths from 562.64 nm to 734.22 nm were tested under the same conditions, with the RH ranging between 40% and 90%. The total wavelength shift increased from 18.74 nm to 50.42 nm and then dropped to 22.63 nm, with the initial wavelength increased from 562.64 nm to 645.52 nm and further to 734.22 nm. Correspondingly, the averaged sensitivity increased from 0.37 nm/RH% to a maximum of 1.01 nm/RH% and then decreased to 0.45 nm/RH%, as shown in Figure 3.

### 3.2. Sensitivity vs. Thickness of PVA Film

**Figure 4:**
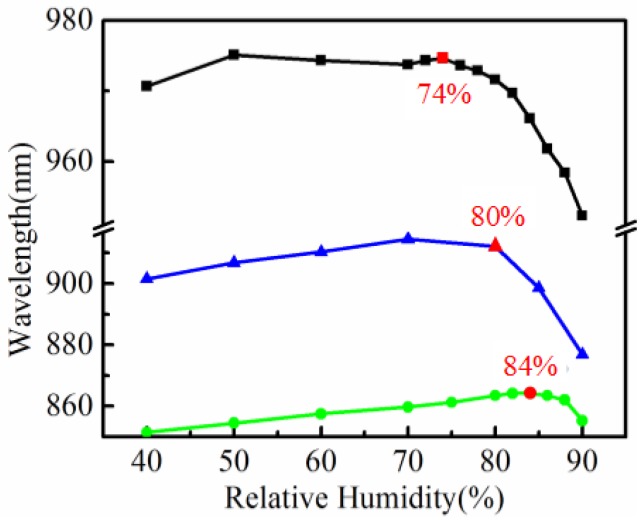
Nonmonotonic shift of the resonant wavelengths with RH increasing for fiber SPR sensors with initial wavelengths of 864.22, 912.10 and 974.64 nm, respectively. The red numbers indicate the inflection points of the sensors.

Further experiments indicate that the shift of the resonant dip will be nonmonotonic once the initial wavelength of the SPR sensor exceeds 850 nm. In these cases, the resonant wavelength will shift to longer wavelengths firstly with the RH increasing, and then shift towards shorter wavelengths with the RH further increasing. There is a certain point of RH, where the wavelength shift meets an inflection, for each sensor with initial wavelengths that go beyond 850 nm. The resonant wavelengths with RH that change from 40% to 90% for three sensors with initial wavelengths of 864.22, 912.10, and 974.64 nm are plotted in Figure 4, respectively. Obviously, inflection points exist for the resonant wavelength shift and read 84%, 80%, and 74% for these sensors, respectively, as a result. And the RH at these inflection points decreases apparently while the initial wavelength of the sensor increases. The RH sensitivities of these sensors before the inflection points are further decreased when compared to those shown in Figure 3. However, sensitivities grow rapidly for the cases of RH that exceed the inflection points. For example, the averaged sensitivity reaches −3.53 nm/RH% in the RH range between 80% and 90% for the fiber SPR sensor with an initial wavelength of 912.10 nm, as can be seen in Figure 4.

The appearance of an inflection point for a resonant wavelength shift of the fiber SPR sensor can be mainly attributed to the competition between the thickness expansion and RI reduction of the PVA film due to moisture absorption in the humidity atmosphere. As is well known, the swelling is notable for PVA films due to moisture absorption [29], which will thicken the PVA film coated on the surface of the fiber SPR sensor and thus result in the redshift of the resonant wavelength of the sensor. Preliminary simulation results show that a 10 nm increment of the film-thickness may result in a resonant wavelength shift toward longer wavelengths for ~15 nm for a 100-nm-thick PVA film. On the other hand, the RI reduction of the PVA film due to moisture absorption may lead to the shift of the resonance toward shorter wavelengths [19]. Specifically, with the film-thickness increasing, the total shift of the resonant wavelength caused by this effect becomes gradually larger, from the order of tens to hundreds of nanometers. So PVA swelling may play a major role in the resonant wavelength shift for fiber SPR sensors with very thin PVA films, which typically exhibit an initial wavelength smaller than 850 nm. In these cases, the penetration depth of the evanescent field is larger than that of the thickness of the PVA film, and a notable fraction of light fields penetrate the film and propagate in the air nearby. For sensors with an initial wavelength larger than 850 nm, the fraction of evanescent fields that propagate in the air decreased, and the two phenomena of PVA film caused by moisture absorption may compete with each other and exhibit a balance at an appropriate point of RH, namely, an inflection point. The resonant wavelength shift is dominated by the film expansion before this inflection point, and then dominated by the RI reduction of the PVA film after this point. Experimental results shown in Figure 4 have proven the existence of the inflection points. It can also be seen from the figure that the inflection point moves to lower RH values with the initial increases in wavelength. And it can be expected that the effect of RI reduction will fully overcome that of the thickness expansion of the PVA film with a further increase to the film thickness, or the initial wavelength of the fiber SPR sensor [26].

**Figure 5:**
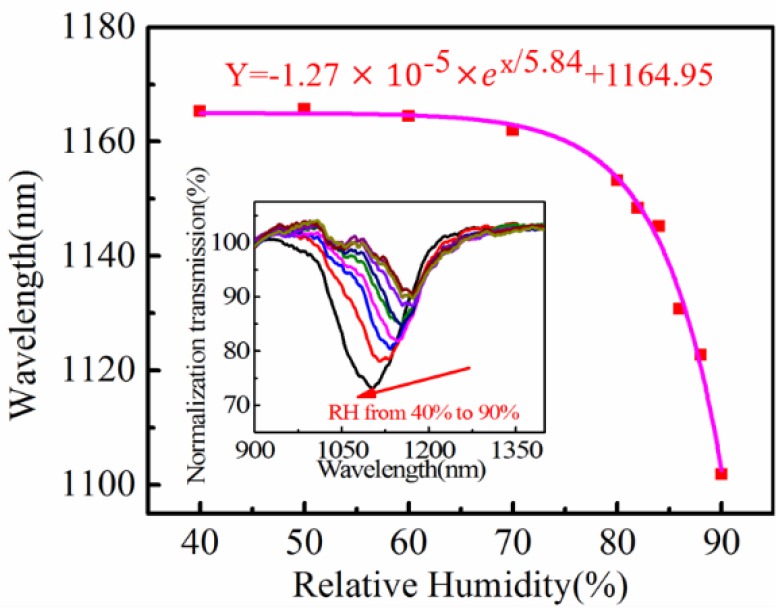
Monotonic shift of the resonance towards shorter wavelengths with RH increasing for a fiber SPR sensor with an initial wavelength of 1165.29 nm.

To verify this expectation, a sensor with an initial wavelength of 1165.29 nm is prepared and tested in the RH chamber. Resonant wavelengths of the sensor are plotted in Figure 5 with RH increasing from 40% to 90%. The resonant dip shifts monotonically towards shorter wavelengths with RH increasing, and can be fitted exponentially with an R-Square of 0.993, as shown in Figure 5. The sensor exhibits very high sensitivity in high RH environments, for example, the averaged sensitivity reaches 4.97 nm/RH% for environments with RH between 80% and 90%.

The sensitivity of different fiber-optic configurations for humidity sensing with wavelength demodulation techniques are list in Table 1, and it can be seen that the RH sensitivity of the device proposed in this work is comparable to the highest reported so far, and the dynamic range of our sensor is also adequately broad. 

### 3.3. Stability

**Figure 6:**
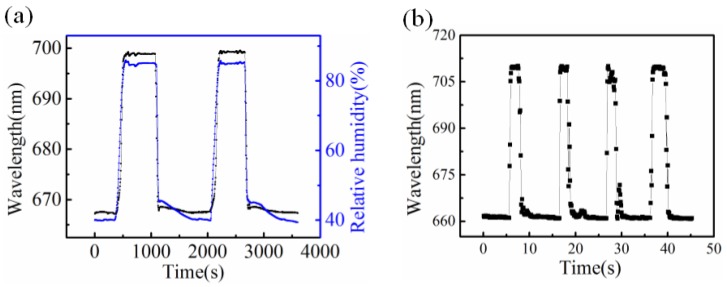
(**a**) Cycling response with RH changes between 40% to 85% and (**b**) human breaths response for a fiber SPR sensor with initial wavelength of 664 nm. The blue curve in (**a**) represents the data acquired from the electric hygrometer for calibration.

The fiber SPR sensor we propose also shows good stability and repeatability under RH cycling test. For the RH cycling test, the sensor demonstrated in Figure 2 (with an initial wavelength of 645.52 nm) is placed in the RH chamber, where the RH is changed from 40% to 85% rapidly and maintained for 10 mins and then dropped to 40% many times. In these cycles of RH iteration, the resonant wavelength is recorded by the spectrometer and PC for every 5 s. Figure 6a shows the wavelength changes of the sensor in two cycles, and results indicate that the sensor exhibits good stability for high RH levels and repeatability under RH cycling test. Figure 6b illustrates the sensor response to human breath, the data of which are obtained by applying periodic breaths onto the sensing area and recorded by spectrometer for every 100 ms. We can see clearly that the resonant wavelength of the sensor shifts to longer wavelengths and goes back immediately with human breaths [30], and the response time (10% base line to 90% maximum) can be calculated to be 251 ms by averaging rising times of the respirations shown in Figure 6b. The RH data obtained by the electric hygrometer are also recorded by PC in real-time and plotted as blue dotted lines in Figure 6a for calibration.

### 3.4. Temperature Cross-Sensitivity

**Figure 7:**
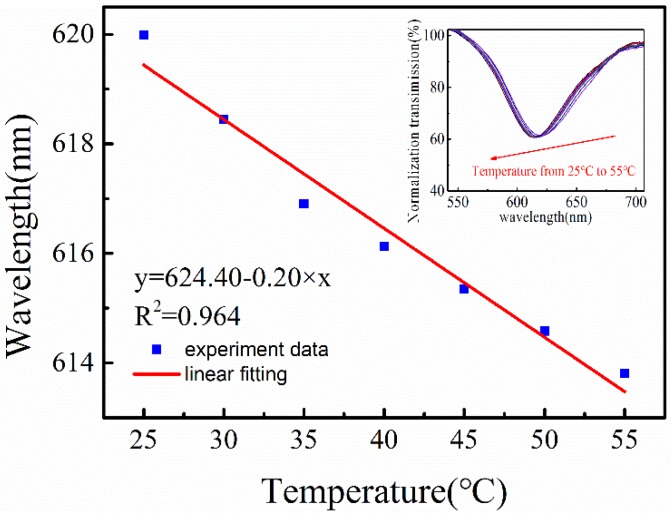
The resonance dip shift of the sensor with the initial wavelength at 619.99 nm when the temperature changes from 25 °C to 55 °C.

The temperature response is shown in Figure 7. When the environmental temperature increases from 25 °C to 55 °C, the SPR wavelength shifts from 619.99 nm to 613.81 nm. A temperature sensitivity of 0.20 nm/°C can be obtained through linear fitting of the experiment data with R-square of 0.964. Accordingly, the temperature induced cross-sensitive can be calculated to be 0.2 RH%/°C.

## 4. Conclusions

We have proposed a simple RH sensor based on a fiber SPR device. The sensor is fabricated by side-polishing a single mode fiber, coating a 55-nm-thick Au film on the polished surface and then dip-coating a thin layer of PVA film onto the Au-surface. The competition effect between the thickness expansion and RI reduction of the PVA film has been investigated, with the proposed fiber SPR configuration under different steady state humidity levels. The resonant wavelength of the sensor in ambient air can be tuned by adjusting the thickness of PVA films. With the initial wavelength increasing from 550 nm to 750 nm, the resonant wavelength redshifts and the averaged sensitivity increases firstly and reaches a maximum of 1.01 nm/RH% and then drops. With initial wavelengths exceeding 850 nm, the resonant dip of the sensor shifts to longer wavelengths firstly and meets an inflection point and then shifts toward shorter wavelengths with increasing RH. And with the initial wavelength of the sensor further increasing, the resonant wavelength shifts toward shorter wavelengths monotonically with increasing RH, and a sensitivity as high as −4.97 nm/%RH can be achieved in this case. The proposed sensor is simple in structure and highly sensitive to humidity, and may find potential applications in the fields of humidity sensing.

## Figures and Tables

**Table 1:** The sensitivity and dynamic range of different configurations.

	Configuration	Sensitivity	Dynamic Range (%RH)
W.C. Wong [3]	Michelson interferometer	0.60 nm/%RH	30% to 90% RH
S. Wu [4]	F-P interferometer	−23.1 pm/%RH	30% to 90% RH
T. Li [5]	PCF-mode interferometer	40.9 pm/%RH	20% to 95% RH
C. Zhao [19]	PVA coated photonic crystal cavity	129 pm/%RH	40% to 90% RH
C.R. Zamarreno [25]	ITO coated optical fiber	0.83 nm/%RH	20% to 90% RH
This article	PVA coated SPR fiber	1.01 nm/%RH	40% to 90% RH
